# Cold chain and severe acute respiratory syndrome coronavirus 2 transmission: a review for challenges and coping strategies

**DOI:** 10.1515/mr-2021-0019

**Published:** 2022-03-01

**Authors:** Jiangtao Liu, Tongzhang Zheng, Wei Xia, Shunqing Xu, Yuanyuan Li

**Affiliations:** Key Laboratory of Environment and Health, Ministry of Education & Ministry of Environmental Protection, and State Key Laboratory of Environmental Health, School of Public Health, Tongji Medical College, Huazhong University of Science and Technology, Wuhan, Hubei, China; Department of Epidemiology, School of Public Health, Brown University, Providence, RI 02912, United States

**Keywords:** cold chain, coronavirus disease 2019, prevention and control, severe acute respiratory syndrome coronavirus 2

## Abstract

Since June 2020, the re-emergence of coronavirus disease 2019 (COVID-19) epidemics in parts of China was linked to the cold chain, which attracted extensive attention and heated discussions from the public. According to the typical characteristics of these epidemics, we speculated a possible route of transmission from cold chain to human. A series of factors in the supply chain contributed to the epidemics if the cold chain were contaminated by severe acute respiratory syndrome coronavirus 2 (SARS-CoV-2), such as temperature, humidity, personal hygiene/protection, and disinfection. The workers who worked in the cold chain at the receiving end faced a higher risk of being infected when they were not well protected. Facing the difficult situation, China put forward targeted and powerful countermeasures to block the cold chain-related risk. However, in the context of the unstable pandemic situation globally, the risk of the cold chain needs to be recognized and evaluated seriously. Hence, in this review, we reviewed the cold chain-related epidemics in China, analyzed the possible mechanisms, introduced the Chinese experience, and suggested coping strategies for the global epidemic prevention and control.

## Introduction

From early 2020, the coronavirus disease 2019 (COVID-19) outbreak globally posed a significant burden to human health and regular social order. So far, the world has faced unprecedented challenges with more than 100 million people accumulatively infected by severe acute respiratory syndrome coronavirus 2 (SARS-CoV-2), while the situation is highly uneven among countries [1]. Effective and safe vaccination is undoubtedly a key measure to fight against the pandemic at the present stage [2, 3]. Increased production and application of vaccines will help the world ease the outbreak and save lives. While, as World Health Organization (WHO) warned in 2020, basic public health measures remain the foundation of the response to pandemic [4]. To contain and end the pandemic earlier, the governments still need to carry out robust public health measures to reduce the transmission in the community, especially when facing unknown risks which might cause a re-emergence of the outbreak.

Since June 2020, the re-emergence of locally transmitted cases in China has aroused people’s great attention, including the epidemics in Beijing [5], Qingdao [6], Dalian [7, 8], all of which have been linked to the cold chain. The common characteristics of these epidemics were that there were no new reported local cases for months before the latest round emerged, and the SARS-CoV-2 contaminated cold chain (such as food or its packaging) was thought to be the source of infections. China introduced a series of strict measures to deal with cold chain-related risks. For example, the government put forward professional guidelines to help standardize the work in prevention and control linked to the cold chain. The situation also prompted a heated discussion about the risks of the cold chain in China.

However, the opinions about cold chain-related risks seem to be controversial in the world. Many countries or organizations think that the infection risk from food is shallow. According to WHO, the SARS-CoV-2 must multiply and survive in a live animal or human host, other than on the surface of food packages [9], and food packaging has not presented any specific risk of transmission. Similar opinions are also held by International Commission on Microbiological Specifications for Foods [10], the United States [11], European Commission [12], and Australia/New Zealand [13]. However, some studies have indicated that viruses may enter industrialized or traditional processing foods, and the contamination risk could be augmented by a complex farm-to-table process [14, 15]. In Europe, several severe outbreaks occurred in slaughterhouses or meat packing plants with favorable environments for SARS-CoV-2 transmission [16], which indicated that the unique environment of the food industry might be suitable for the survival and spread of the virus. Based on the discoveries of SARS-CoV-2 on cold chain food packaging in some countries, Vietnam also demanded that samples collected from imported food packaging, especially frozen foods, should be tested for SARS-CoV-2 [17]. Even though it’s controversial, considering the unstable epidemic situation globally and the cold chain-related epidemics in China, the risks should not be ignored. More importantly, the recent report of WHO-convened global study of origins of SARS-CoV-2 has also indicated a possible SARS-CoV-2 transmission route via frozen products [18]. Thus, further analysis and studies need to be conducted to explore the potential risk of the cold chain.

In this review, based on the cold chain-related epidemics in China, we analyzed the potential risks of SARS-Cov-2 transmission in the cold chain systematically, and introduced targeted strategies to minimize the transmission possibility and risk, which would have important public health implications for the world to contain the pandemic effectively.

## SARS-CoV-2, COVID-19 and its infectivity

The SARS-CoV-2 is the seventh coronavirus known to cause infections in humans [19, 20]. Among the seven coronaviruses, HKU1, NL63, OC43, and 229E could lead to mild symptoms in humans [21], while the SARS-CoV, Middle East respiratory syndrome coronavirus (MERS-CoV, and SARS-CoV-2, all identified in the past 20 years, could cause severe diseases, including severe acute respiratory syndrome (SARS, 2003), Middle East respiratory syndrome (MERS, 2012), and COVID-19, respectively [19, 22]. SARS-CoV-2 is an enveloped single-stranded RNA virus and covered by a lipid bilayer. The envelope protein spike (S) presents as a trimer on the surface of SARS-CoV-2 and facilitates entry of the virus into host cells [23]. As a target for viral infection, the host angiotensin-converting enzyme 2 (ACE2) distribution is a crucial factor for determining viral tropism [24]. Some previous studies have shown that the viral load of newly infected patients increased to a higher condition in the upper and lower respiratory tracts successively, further indicating that the upper respiratory tract is the usual initial site of viral replication [25, 26]. For humans, susceptibility to SARS-CoV-2 infection increases with age, and children seem to be less susceptible than adults [27].

Environmental factors, including climatic change, deterioration of ecosystems, extreme weather, play a formidable role in influencing the trends of viral infections [28]. COVID-19 pandemic emerged in winter before the subsequent worldwide spread and aggrandized in the winter, indicating the possible seasonality [29, 30]. Many previous studies have also pointed out that environmental factors, such as meteorological variables, air pollution, ultraviolet (UV), may influence SARS-CoV-2 transmission and development [31], [32], [33], [34], [35]. Meteorological factors or possible seasonality could affect the host vulnerability through moderating innate defense mechanisms and thus, may regulate the resistance to viruses. Unlike bacteria, viruses could only survive in the host cell, which may not reproduce in a food environment [36, 37]. However, food may play a role in the transmission of viruses to humans. Some previous studies have indicated that the food could be contaminated through several different routes, including contamination during food processing, direct contamination of human feces, contaminated food animal origin, and inadequate wastewater treatment [15, 37], [38], [39]. The above environmental factors could affect transmission intensity, persistence in the environmental matrices (including food contact surfaces or packaging materials) [15], and infection potential [40]. Besides, these environmental factors could influence the maintenance of virus viability on the different object surfaces [41].

## The evidence for cold chain-related epidemics

### The cold chain-related epidemics in China

After winning the battle for the severe outbreak in Wuhan in early 2020, China’s efforts for epidemic prevention and control have turned to regular epidemic prevention and control. The locally transmitted epidemics in Beijing, Qingdao, Dalian, and Tianjin happened unpredictably (Table 1). The subsequent epidemiological investigation and sequencing results of the virus indicated the critical roles of the cold chain, which have brought new changes and challenges for the epidemic prevention and control in the stage of new normal in China.

**Table 1:** A summary of the epidemics related to cold chain in China in 2020.

Month	City	Number of infected cases	Possible source of infection
June	Beijing	335	Frozen salmon
September	Qingdao	2	Frozen cod outer packaging
November	Jiaozhou (Qingdao)	2	Aquatic products
July	Dalian	118	Imported seafood processing workshop
December	Dalian	83	Bulk goods of Russian cargo ship
November	Tianjin	10	Imported cold chain food

#### Beijing

**Figure 1:**
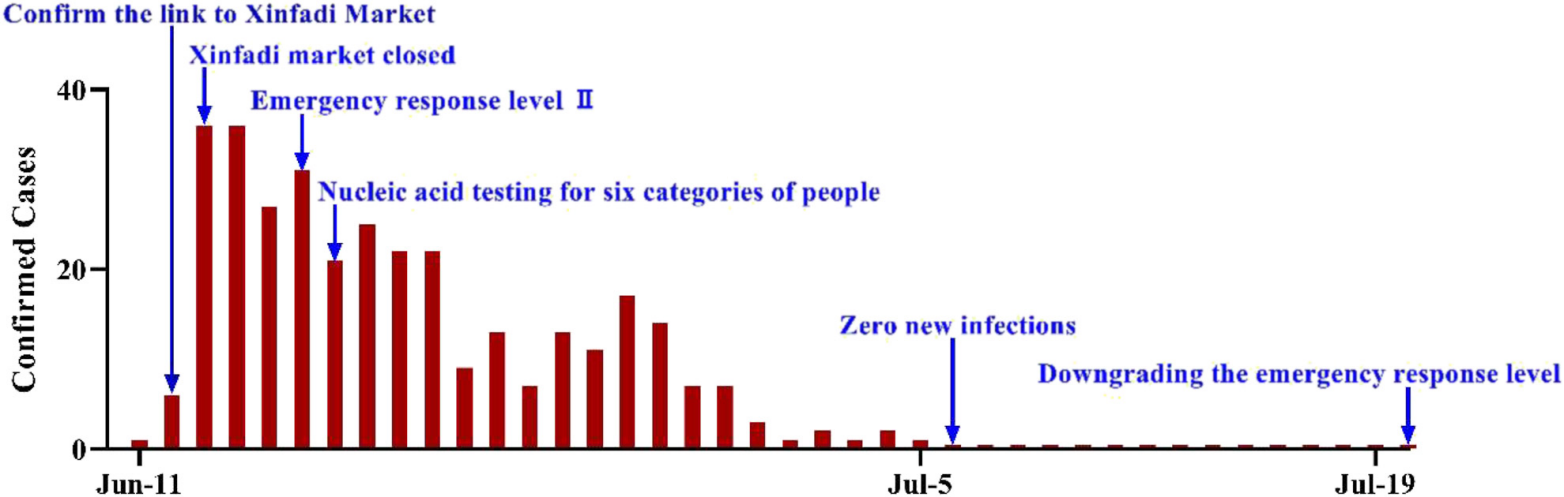
Locally transmitted epidemic development of Beijing Municipality, China in 2020.

In June 2020, after 56 days without reporting new locally transmitted cases in Beijing, a new round of outbreak occurred unexpectedly (Figure 1). In this round of local outbreak, Beijing reported a total of 335 COVID-19 confirmed cases, and epidemiological investigation indicated that the local cases of this wave were linked to the largest wholesale market in Beijing, called Xinfadi market. It’s awe-inspiring that Beijing acted rapidly after the new local case report on June 11. Xinfadi wholesale food market was identified as a direct source of infection within 22 h which gained valuable time for further measures. Due to the rapid response, the number of daily new cases fell to zero after approximately one month, and subsequently, the municipal government decreased its COVID-19 emergency response level.

After sequencing the samples from patients and the environment in the Xinfadi market, the virus causing the outbreak was confirmed to belong to the L lineage European branch I indicating the possibility of virus transmission from overseas. Through a thorough investigation, the origin of this outbreak was verified to link with the supply of the contaminated salmon in booth #S14, and early patient exposure happened on May 30 before the first case was reported [5, 42]. Pang et al. further speculated that the COVID-19 resurgence in Beijing was likely initiated by an environment-to-human transmission originating from contaminated imported food via cold chain logistics [5].

The outbreak had been a cause of concern that whether the outbreak would develop into a second wave nationwide after the outbreak in Wuhan at that time. Thankfully, this has not happened. Due to the problematic epidemic situation, Beijing has taken robust measures to respond to the outbreak, such as conducting massive nucleic acid testing, upgrading emergency response levels, appropriate lockdown. It is worth mentioning that massive nucleic acid testing has played a crucial role in achieving final victory over the outbreak.

#### Qingdao

The epidemic in Qingdao was a typical and convictive instance in affirming the existed transmission possibility from cold chain to human. On September 24, 2020, two asymptomatic infections were reported by Qingdao [43]. The cases were identified in Qingdao Port when they underwent the routine nucleic acid inspection. They were two stevedores who had close contact with imported cold chain food in daily work. Epidemiological investigation showed that both cases had no intimate contact history with COVID-19 case or travelers from overseas [6]. In the process of tracing, positive SARS-CoV-2 nucleic acid was detected in the samples collected from the frozen cod outer package. Further analysis showed that the virus contributing to the outbreak belonged to the strain of the European branch (L lineage B1.1), which also originated from Europe [6]. The Chinese Center for Disease Prevention and Control (CDC) isolated SARS-CoV-2 from the imported frozen cod outer package’s surface for the first time, and the finding confirmed that the SARS-CoV-2 virus could survive after a long journey in the cold chain [6]. Hence, the epidemic in Qingdao was probably caused by SARS-CoV-2 contaminated cod outer package during production or cold chain transportation [6]. And then, in October, the source of a cluster outbreak was traced to the epidemic in September, which spread through a CT scanning room shared by the two COVID-19 infections and those with other conditions at a hospital. It also verified that SARS-CoV-2 could survive on the surface of medical settings, and complete disinfection is necessary for the risky areas, especially in the medical settings.

At the end of November 2020, a stevedore who worked in a fishery company was confirmed as an asymptomatic COVID-19 case in Jiaozhou, Qingdao, through the routine nucleic acid inspection. A co-resident who was the close contact of the case was also infected. Cold chain samples from the fishery company tested positive for SARS-CoV-2, and the traceability results confirmed that the SARS-CoV-2 contaminated cold chain food caused the epidemic [44].

#### Dalian

**Figure 2:**
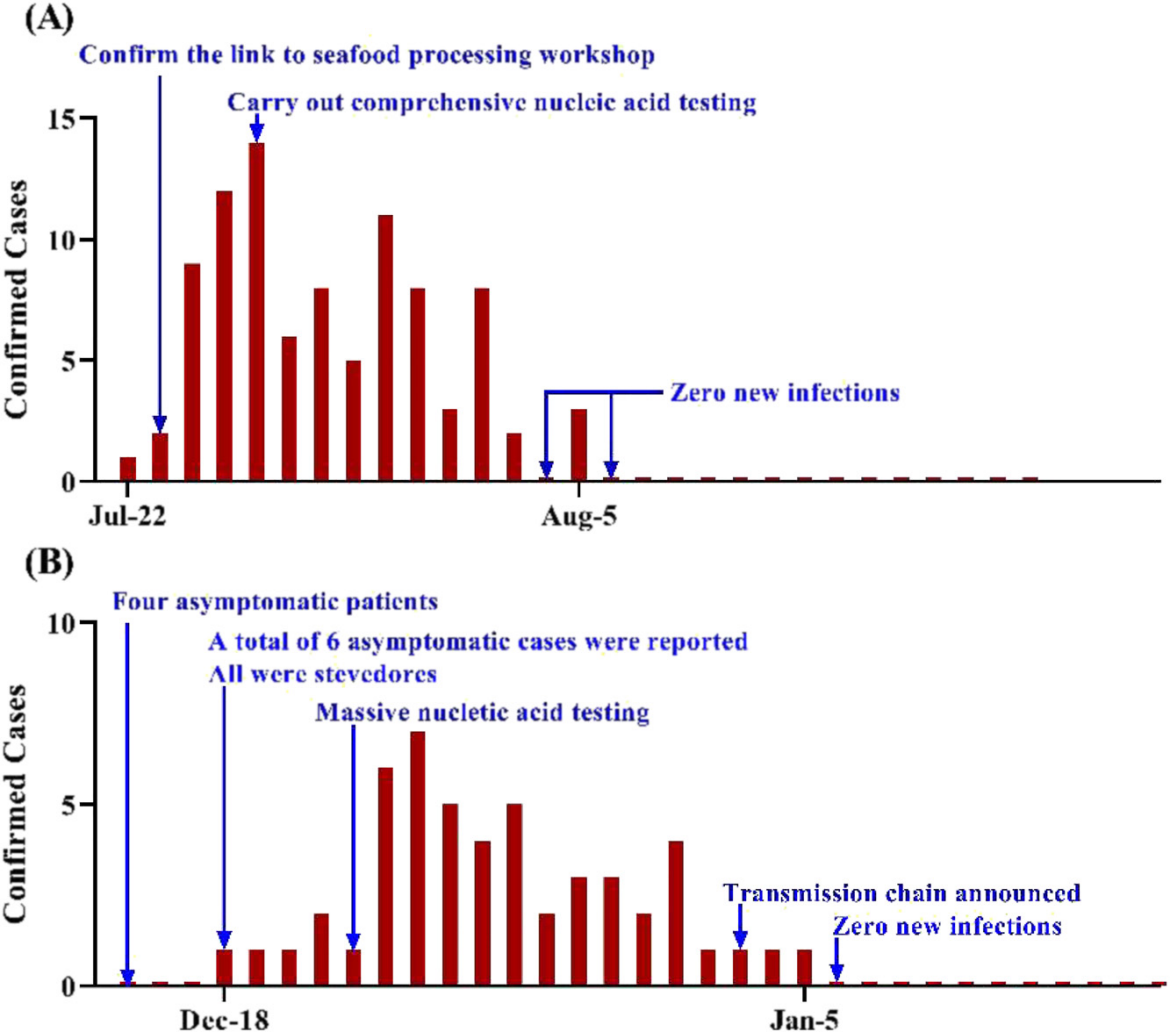
Locally transmitted epidemic development of Dalian, Liaoning Province, China in 2020. The figures only present confirmed cases. (A) The first round in July, 2020; (B) The second round in December, 2020.

In July and December 2020, two waves of epidemics linked to the cold chain occurred in Dalian, Liaoning province [45] (Figure 2). It is reported that most cold chain foods imported to Dalian were from high-risk areas, which is a significant threat.

The first wave lasted from July to August 2020, and 118 local COVID-19 positive infections (92 confirmed cases, 26 asymptomatic infections) were reported. There was no clue showing that the epidemic in Dalian was related to the local cases in domestic (Beijing, Xinjiang, etc.) through the method of case investigation and big data comparison. Further investigations indicated that this cluster outbreak was mainly related to a local, imported cold chain seafood processing workshop. All cases belonged to the same transmission chain due to the highly homologous virus. According to the official source, the outbreak started at the seafood processing workshop and spread to other places [46].

The second wave occurred in December, and a total of 83 locally infected cases were reported. Among the infections, 51 were confirmed cases, and 32 were asymptomatic infections. The earliest infections in the outbreak were dockworkers at Dalian Port who were infected by SARS-CoV-2 contaminated cold chain bulk goods on Russian cargo ships, and then the activities of the dockers caused a wider spread [7]. The genome sequencing results indicated that the strain collected from the patient was highly similar to the one in the imported cold chain products, which was also the one prevalent strain in Russia in November 2020 [47]. Due to the two-round locally transmitted epidemics, to cut off the transmission of COVID-19 from overseas, the port city Dalian has maintained precautions for imported cold chain foods [48].

#### Tianjin

In November 2020, North China’s Tianjin Municipality confirmed domestically infected COVID-19 cases on the two separate transmission chains [49]. A total of 10 people got infected by the virus. The source of COVID-19 infection of the two chains occurring in the early winter was both imported cold chain food. People working in the cold chain were the first to be infected. The transmission chain that happened in a community spread more widely due to poor personal protection and personal hygiene. The virus genotypes of the two chains were L-lineage European branch I and branch Ⅱ.

### The evidence out of China

In 2020, a new cluster in New Zealand was reported after 102 days without any local transmission [50]. One of the infected persons was involved in handling frozen foods, and he was on sick leave for nine days before the time he tested positive [50]. The infections have aroused discussion about whether frozen goods were the source in New Zealand.

In Europe, many outbreaks have been reported in slaughterhouses and meatpacking plants [16]. Since May 2020, several outbreaks in the German meat industries have occurred despite various protective measures [51, 52], such as the outbreak in North Rhine-Westphalia [16]. As mentioned in the previous editorials [16], similar outbreaks have also been found in Portugal, England, and Wales. These outbreaks were partly due to the favorable environments for virus transmission. In the United States, the outbreaks among meat and poultry processing facilities rapidly affect many persons [53], [54], [55]. Due to the uneven development of global epidemics, different countries have implemented different prevention and control measures. The cold chain-related outbreaks have not been reported directly, and only a handful of countries have carried out nucleic testing for SARS-CoV-2 in cold chain-related food/packaging or environment. These outbreaks in Europe and the United States were partly similar to the epidemics in the cold chain in China.

## Transmission characteristics of cold chain-related epidemics

The transmission of infectious diseases (including COVID-19) must rely on three primary links: infection source, transmission route, and susceptible population, which could play a decisive role in the occurrence, transmission, and termination of infectious disease [56, 57]. The cold chain-related epidemics are no exception, and some common characteristics exist among these events.

### Infection source

The contaminated cold chain might be the chief culprits of epidemics in these Chinese cities mentioned in the previous section. Among the cities suffering from cold chain-related epidemics, most of them were port cities where received imported cold chain food frequently and directly, such as Tianjin, Dalian, and Qingdao. The epidemics all happened in the cold chain, including market, refrigeration house, food processing workshop, which could partly support the speculation about the role of the cold chain in the introduction of SARS-CoV-2. Further, Joint WHO-China Study has also shown that infection from cold/food chain products is a possible pathway in Wuhan, China [18].

The imported cold chain products might be contaminated by the infected workers from severely affected areas through respiratory droplets and fecal shedding [58], [59], [60], [61], [62]. Virological analysis for COVID-19 patients shows that the virus replicates actively in the tissues of the upper respiratory tract and pharyngeal virus shedding is very high during the first week of symptoms [25]. The SARS-CoV-2 from active pharyngeal viral shedding could be infectious [25, 63]. If the pre-symptomatic or asymptomatic cases don’t achieve regular nucleic acid testing, the virus might be spread widely without even knowing it. Besides, as a zoonotic virus, the consumption of animal-based products might bring the risk [38].

If the markets or cold chain workshops don’t maintain standard practices at the receiving end, the virus might jump the species barrier and cause infection among workers. For example, in Xinfadi Market, specific environmental factors influenced the dissemination of viruses across the market [42]. Hence, the whole supply chain might be risky for the spread of the virus.

### Transmission routes

Like many other respiratory infectious diseases, the most common SARS-CoV-2 human to human transmission mode is droplets expelled during face-to-face talking, coughing, or sneezing [19]. Surface contact is another possible transmission mode when contaminated heavily by the virus [19]. Besides, the transmission may also occur via aerosols (smaller droplets that remain suspended in air) [19, 64, 65]. Although the above are the main routes for SARS-CoV-2 transmission, there are some other modes discussed hotly. The transmissions could occur through these routes in different scenarios, such as in households, animal farms, or public facilities [66]. Superficially, it seems that the cold chain-related epidemics transmit in a route from cold chain to human. In effect, combining the fact that cases in the early epidemic stage have no histories of overseas travel, close contact, or other known risky behaviors related to SARS-CoV-2 transmission, the cold chain-related SARS-CoV-2 transmission might be an indirect transmission (fomite transmission). Some previous studies have suggested that indirect contact transmission is the predominant transmission route for some respiratory viruses (including influenza) in some settings [67], [68], [69]. The cold chain-related epidemics will undergo the process of “thing to human” and “human to human”, and different transmission routes contribute to the epidemics in various stages.

In general, indirect contact transmission occurs by the passive transfer of microorganisms to a susceptible host via an intermediate object, such as contaminated surfaces around the patient’s immediate environment [68]. Contaminated surfaces are reported to act as significant vectors in the survival and transmission of many viruses, including coronaviruses, influenza viruses, paramyxoviruses, poxviruses, and retroviruses [70], [71], [72], [73], [74]. Indirect contact transmission is a route by touching a surface with coronavirus contaminated, which has also been referred to as fomite transmission [19, 75, 76]. An investigation in Wenzhou, China, has shown that the rapid spread of SARS-CoV-2 in a shopping mall resulted from spread via fomites (e.g., elevator buttons or restroom taps) [77], similar to the situation in Tianjin [49]. For cold chain logistics, the lockdown adopted by the national governments could stop the contact from human to human, while indirect transmission might occur through shared contact of contaminated objects [75]. If the virus keeps alive after the transportation, the workers at the receiving end might contact fomites and then be infected by touching their mouth and nose, which gives the virus a chance to get into the body. Besides, some studies indicate that the virus also has the potential for viral entry from the human eyes [78]. It’s worth mentioning that the live virus on human skin could survive more than 9 h and might increase the risk of human to human transmission [79]. The direct contact of SARS-CoV-2 contaminated surface contributes to the infection of workers, and the workers might spread the virus a broader scope during the process of daily activities. Combined with the characteristics of zero patients of these epidemics, fomite transmission leads to the occurrence of “thing to human” transmission. However, fomite is far less efficient in spreading SARS-CoV-2 [80], which could partly explain why only a handful of cities report cold chain-related epidemics despite a large number of positive reports in the cold chain.

Respiratory droplets and aerosols, produced during various expiratory activities, such as speech and coughing, might contain viral particles remaining viable and infectious [81, 82], especially in the poorly ventilated spaces [82]. The confined and populated spaces, like public transport and nursing homes, have been reported as sites of viral transmission despite preventive physical distancing [82]. It should also not be ignored that persons in close proximity in indoor settings with poor ventilation for extended periods might contribute to the superspreading events [26]. Cold chain-related environments, such as seafood markets and logistics stations, are comparatively confined and crowded, facilitating the easy transmission of viruses. Such an environment even prolongs or enlarges the chance for workers to contact the virus, thus expanding the possibility of infection, which are the favorable conditions for the “human to human transmission.”

### Susceptible population

Susceptibility should be considered in the workplace due to the complex interplay between individuals and the job or task features [83]. There have been many outbreaks and clusters of COVID-19 in various occupational settings since the start of the pandemic reported in Europe [84]. Workers might have to keep close physical proximity to other people (co-workers, patients, customers, etc.) in occupational settings. Without mitigation measures, the risk could be higher, particularly in indoor environments or with shared transport or accommodation [84]. We can speculate that, at the receiving end of cold chain products, the people, especially those workers who contact the contaminated cold chain directly and repeatedly, face a higher risk of being infected if they don’t protect themselves well. In fact, the patient zero of these cold chain-related epidemics were the workers, and further, the virus spread among humans in the community in some cities. The biological risks in the cold chain are intrinsically related to job activities [83]. One important feature is that these workers must contact the cold chain frequently in their daily work, which raises their risk of infection. Above all, the work in the cold chain can impact the infection risk, and demographic and pathological characteristics of workers can influence COVID-19 severity [85], which urges better protection for cold chain-related workers. These workers should be regarded as high-risk people in the process of epidemic prevention and control.

To sum up, the cold chain presents to be a risky source of the SARS-CoV-2 infection, which might further cause an outbreak in clusters or even community transmission (Table 2).

**Table 2:** A summary for the basic links in the cold chain-related transmission and outbreaks.

Basic links	Concrete contents
Infection source	Cold chain (frozen food/packaging/workplace)
Transmission routes	Indirect transmission (fomite transmission)
Susceptible population	Cold chain-related staff (stevedore, dock-man, food processing worker, etc.)

## The risk analysis for cold chain-related spread

SARS-CoV-2 contaminated cold chain doesn’t necessarily lead to human infection, which relates to several factors, such as viral load, virus viability, and protection measures. It is essential to recognize and evaluate the cold chain-related risk in epidemic prevention and control. This section will discuss the factors influencing the risk and present a reference for coping strategies.

### The virus viability in the environment

The lifespan, diffusion, migration, and variation of infectious of SARS-CoV-2 could be influenced by various environmental factors, including temperature, humidity, UV radiation, etc. [86]. For example, temperature is a critical factor that can directly affect the viability of coronavirus in the environment and on nonliving surfaces [87]. Previous studies have explored the SARS-CoV-2 stability and viability on various surfaces under different environmental conditions [81, 88], [89], [90], as shown in Table 3. The virus remains viable and infectious in aerosols for hours and on surfaces up to days, depending on different laboratory conditions [81, 90, 91]. Compared with the virus on copper and cardboard, SARS-CoV-2 virus is more stable on plastic and stainless steel [81]. The SARS-CoV-2 could survive up to 28 days on glass, steel, and polymer, paper banknotes [90, 91]. The viability of SARS-CoV-2 on the lifeless surface is similar to SARS-CoV [92], which could expand the infection risk through the contaminated surfaces in the cold chain. The viability and spread of SARS-CoV-2 are associated with environmental conditions, and indeed, low temperatures and humidity could prolong the survival of the virus regardless of surface type [90, 93, 94]. It is shown that the virus is highly stable at 4 °C, whether the virus is in dried form or solution [91, 95], but sensitive to heat, after comparing virus titer change at 4°C and 70 °C [91]. In another study, it has been indicated that the virus is more stable at relatively low relative humidity (RH) and <24°C [93]. Besides, SARS-CoV-2 is highly durable in a wide range of pH values from acidic to basic environments at room temperature [91, 96].

**Table3:** The SARS-CoV-2 viability under different environmental condition in laboratory.

Surface/solution/material	Temperature/humidity (RH)	Outcome indicator	Outcome	References
Aerosols	21–23°C, 65% RH	Half-life (h, 10^5.25^ TCID_50_/mL)	1.09 (95% CI: 0.64, 2.64)	[81]
Cardboard	21–23°C, 40% RH	Half-life (h, 10^5^ TCID_50_/mL)	3.46 (95% CI: 2.34, 5)	[81]
Steel	21–23°C, 40% RH	Half-life (h, 10^5^ TCID_50_/mL)	5.63 (95% CI: 4.59, 6.86)	[81]
Plastic	21–23°C, 40% RH	Half-life (h, 10^5^ TCID_50_/mL)	6.81 (95% CI: 5.62, 8.17)	[81]
Glass and banknote	22°C, around 65% RH	Virus titre (Log TCID_50_/mL)	Undetectable on day 4 (5.83 and 6.85 log-unit on day 0)	[91]
Stainless steel and plastic	22°C, around 65% RH	Virus titre (Log TCID_50_/mL)	Undetectable on day 7 (5.80 and 5.81 log-unit on day 0)	[91]
Stainless steel, ABS plastic, and nitrile glove	24°C, 20% RH	Half-life (h, ±SD)	15.33±2.75	[93]
	24°C, 40% RH	Half-life (h, ±SD)	11.52±1.72	[93]
	24°C, 60% RH	Half-life (h, ±SD)	9.15±3.39	[93]
	24°C, 80% RH	Half-life (h, ±SD)	8.33±1.80	[93]

SARS-CoV-2, severe acute respiratory syndrome coronavirus 2.

As we know, in the cold chain logistics distribution, cardboard, plastic, metal, glass, etc., are the primary packaging materials used, and plastic is the most used. Thus, the above mentioned studies indicate that the virus might survive long on the surfaces of the cold chain, which provides a SARS-CoV-2 favorable environmental condition to stay long and even keep high infectivity.

### Transmission risk

Viral infectivity in the cold chain might be influenced by many factors, including the viral load absorbed on the environmental surfaces [97]. Although the cold chain-related epidemics in China have aroused people’s concern about whether there is a significant risk of being infected by the cold chain products in daily life, so far, there have been no consumers reported getting infected due to buying cold chain products. As WHO indicated, there is currently no evidence that people can get COVID-19 from food, including fruits and vegetables [9], and there is also no evidence that COVID-19 is a foodborne disease [92]. The infectivity of viruses in various matrices could lose at relatively high temperature during food processing [98]. Besides, the transmission from contaminated surfaces to humans must meet a set of conditions, including a respiratory pathogen shed into the environment, the capacity to survive on surfaces, transferred to hands or other equipment with the infectious dose, and ability to initiate infection through contact with the eyes, nose or mouth [99].

Many places in China have reported positive coronavirus strains in a cold chain environment [100], while not all positives trigger the infections. It can be speculated that positive nucleic acid on the cold chain products or their packaging does not indicate the infectivity of the virus, and even the presence of a live virus does not necessarily mean it could infect people. The risk is also related to the virus load, even if the virus could survive in the cold chain. As Table 4 shows, real-time RT-PCR cycle threshold (Ct) is an indicator that could reflect viral load in the samples, and lower Ct values are associated with a higher probability of a positive viral culture [101, 102]. A previous study showed that 86.3% of respiratory tract samples tested positive with a Ct value below 30 were culture-positive. In contrast, only 8.3% RT-PCR positive samples with a Ct value over 35 were culture-positive [103]. Lower Ct values may also be associated with worse outcomes in clinical practice [104]. There are some studies presenting virus load of the samples collected from the indoor or outdoor environment. Using the samples collected from hospitals, the samples with the viral RNA detected positively owned high PCR Ct values from various points and were negative for viral culture [105, 106]. Identified viral RNA levels on environmental surfaces of quarantine hotels and used dining utensils were markedly lower than in the nasopharynx of source patients [26, 107, 108]. For the imported cold chain products in China, most of those with positive nucleic acid had high Ct value indicating the low virus burden and infectivity [109]. Besides, the positive nucleic acid on the surface of the cold chain might represent the fragments of RNA belonging to the SARS-CoV-2 virus, which do not imply infectivity of the pathogen [110].

**Table 4:** Examples for SARS-CoV-2 contamination on the different surfaces reported.

Areas	Sampling sites	Samples	Positive samples	Ct value	Related conclusions	References
Healthcare setting	Surface and air	Air sampling, swabbing	23/218; 2/31	>30	The virus would not be culturable.	[105]
Hospital isolation areas.	Preprocessing disinfection pool	Sewage	4/4	29.37, 30.58, 32.42, 33.55	No viable virus was detected by culture.	[106]
Barcelona buses and subway trains	Surface swabs; air conditioning (a/c) filters; air conditioning dust; ambient air	Swabbing samples	30/82	–	The detection of fragments of RNA does not imply infectivity of this pathogen.	[110]
Emergency Department	Emergency department patients care and non-patient care areas; personal protective equipment	Sterile premoistened swabbing	10/192	>35	Surfaces and equipment contamination is low in emergency department.	[112]
Hospital	Environmental surface	Wet swabbing	9/50	>30	Handles, cupboards, light switches, and door handles were positive for the presence of SARS-Cov-2.	[113]
Quarantine room	Bathroom, Bedroom, Living room, Cotton, Ceramic, Metal, Wood, Plastic	Sterile polyester-tipped applicator	28/71	Bathroom: 26–37; Bedroom: 29–38; Living room: >40; Cotton: 28–38; Ceramic: 26–33; Metal: 32–33; Wood: 35–36; Plastic: 32–37	SARS-CoV-2 Environmental contamination distributes widely during the incubation period.	[114]
Quarantine hotel environments	Environmental surfaces	Swabbing samples	18/271	Median: 35 (IQR: 34–36.5)	Moist surfaces were vulnerable to remaining SARS-CoV-2 RNA positive.	[115]

Ct, cycle threshold; SARS-CoV-2, severe acute respiratory syndrome coronavirus 2; IQR, interquartile range.

Hence, the cold chain-related risk should not be ignored. In the transmission dynamics of COVID-19, contact frequency, contact intensity (duration, intimacy, indoor/outdoor location, etc.) play a role [111]. In such conditions, the workers in the cold chain might be infected with long-term repeated contact and heavy viral load.

## Targeted prevention and control measures

Considering that the risks cannot be ignored, strict prevention and control measures covering personal protection, disinfection, vaccination should be put forward and adopted in the whole cold chain products supply chain.

### Personal protection

According to the WHO, the highest hygiene standards should be followed during food safety practices in food premises [116]. The good staff hygienic practices cover the following aspects: proper hand hygiene, frequent use of alcohol-based hand sanitizers, good respiratory hygiene, regular cleaning/disinfection, avoiding close contact with anyone with respiratory illness symptoms [116]. The standards also hold for the workers in the cold chain products supply chain. Wearing masks and proper hand hygiene are the necessary procedures to avoid transmission. Hand hygiene with soaps or hand sanitizers is the first-line defensive measure to fight against COVID-19 [117].

In the workplace of the cold chain, due to the poorly ventilated and confined space, the physical distance between fellow workers with at least 1 m has been recommended by WHO [118]. It might be hard for many places to keep the required distance due to the constraints of conditions and reality. In this case, some alternative measures need to be carried out, such as providing complete personal protective equipment, restricting the number of workers, reducting contact chances. The workers should consider wearing protective gowns and gloves when professionally handling cold chain products [119]. Besides, the workers also need to be given regular training on COVID-19 basic knowledge and usual prevention and control, especially in the cold chain, which can help eliminate or reduce the risk.

For consumers, physical distance, wearing face masks, proper hand hygiene are still effective measures to fight against COVID-19. Other personal protective equipment, such as disposable gloves and goggles, should be applied on time as occasion requires. Nevertheless, consumers who contact cold chain must adopt proper and necessary personal hygiene to prevent possible risk during the pandemic, even though the risk is low.

### Disinfection

The correct disinfection could minimize the transmission risk in each stage of cold chain logistics. The cold chain-related workshops need to be disinfected to avoid transmission, and the workers need to follow a standardized workflow on the premise of good personal hygiene. Similar to other types of coronavirus, SARS-CoV-2 is an enveloped virus with a fragile outer lipid envelope that is more vulnerable to disinfectants [120]. Several factors need to be considered for selecting suitable disinfectants, including the concentration, contact time, the compatibility of the chemical disinfectants and surfaces to be tackled, toxicity, ease of use, and stability of the product [121]. At present, the most used disinfectants for SARS-CoV-2 include alcohol disinfectants, chlorinated disinfectants, peroxide disinfectants, UV, ozone [122, 123]. The specific ingredients and applications of various disinfectants are shown in Table 5. Most of these disinfectants could inactivate SARS-CoV-2 by oxidizing RNA and protein layer [122]. Among these disinfectants, chlorine-based products, especially sodium hypochlorite products with different levels of concentration, could be available for use in a variety of settings. However, potential side-effects of chlorine-based products should be paid attention to due to the rapid inactivation of the organic material. Of course, many other choices could be used in the cold chain, which could be chosen according to the needs and actual conditions.

**Table 5:** The selection of disinfection methods in the cold chain [121, 124].

Disinfectants	Ingredients	Applications
Chlorine-based products	Liquid (sodium hypochlorite), solid or powdered (calcium hypochlorite) formulations	Use in a variety of settings (Object surface, fruits and vegetables, tableware, etc.)
Alcohol disinfectant	Ethyl alcohol	Hand and skin disinfection; object surface disinfection
Peroxide disinfectant	Hydrogen peroxide; peracetic acid	Object surface disinfection; air disinfection
Quaternary ammonium disinfectant	Quaternary ammonium chloride; Quaternary ammonium bromide; etc.	Object surface disinfection
UV	UV ray	Object surface disinfection; air disinfection
Ozone	Ozone	Object surface disinfection; air disinfection

UV, ultraviolet.

## Strategies and measures of China

Due to the unstable situation globally and the potential risk of infection from the cold chain, China has already focused on preventing cold chain-related epidemics and launched guidelines to guide epidemic prevention and control. The guidelines involving the food cold chain include “Technical Guideline for Prevention and Control of Novel Coronavirus in the Production of Cold Chain Food” and “Technical Guideline for Disinfection of Novel Coronavirus in Cold Chain Food Production Processes” [123]. To minimize potential risks, the government has taken a strict and full range of measures (full-chain, closed-loop, traceable management of imported cold chain foods) [124]. The targeted measures adopted by China mainly include the following aspects: protection of cold chain-related workers, disinfection, tracing and managing cold chain imports, and emergency response plan.

### Protection of cold chain-related workers in China

The government requires the producers and operators to adjust and update workers’ health management systems and add management measures for COVID-19 prevention and control. Workers should also be professionally trained to use protective suits (protection suits, face masks) to protect themselves well in the work environment. Another critical measure is the publicity of prevention and control knowledge for workers, including proper personal hygiene. During loading, unloading, and storage, in the premise of well personal hygiene, stevedores need to use suitable personal protective equipment (PPE) to avoid frequent contact with the cargo surfaces. The development and applications of vaccines is one of the most successful and cost-effective health interventions when facing the challenges of infectious diseases in history [125]. COVID-19 vaccines offer hope for the world to end the pandemic as soon as possible [126]. Starting from Dec 15, 2020, China officially launched the COVID-19 vaccination program targeting several vital groups. Cold chain-related workers have already been vaccinated as priority groups who face a higher risk from infection [127, 128].

### Disinfection in China

China has launched professional guidelines for the disinfection of cold chain food production processes [123]. During production, processing, storage, or transportation, regular disinfection in each stage has been stressed. China is also committed to the research and application of new disinfection technologies. For example, the Chinese CDC has successfully developed a formula of chlorinated cryogenic disinfectant with the characteristics of the simple production process, low cost of raw materials, and reliable disinfection effect at low temperature [129]. Recently, China National Nuclear Corporation has developed a study about irradiation disinfection for imported cold chain products contaminated by SARS-CoV-2, and a completed simulation experiment has achieved remarkable progress [130]. Compared with other disinfection technologies, irradiation disinfection owns prominent advantages, including no drug residues, intense ray penetration, thorough disinfection, which might be widely applied in the field of cold chain in the future [130].

### Tracing and managing cold chain imports in China

China has established a national platform to trace and manage cold chain imports. The platform has been put into operation as one of the critical measures to prevent the transmission risk of SARS-CoV-2 infections from the cold chain [131]. China has required the countries of origin and manufacturers to ensure the safety of cold chain products. The imported cold chain food must own the information about food safety and epidemic prevention testing, and the information should be supplied to each link in the logistics chain. The whole chain informative tracing enables accurate traceability and positioning when the cold chain products have been reported for nucleic acid positive. Meanwhile, given the potential risk, Chinese authorities have intensified nucleic acid testing over cold chain shipments and halted imports from higher risk regions [131, 132].

## Implications for global food supply chain

A previous study has summarized four significant issues that should be addressed in the food industry and the food supply chain [133]. Among the four issues, food safety is a significant issue for producers, retailers, and consumers [133]. Every link in the chain of farmland to the dining table is related to food safety, and in the final stages of the food supply chain, more people who might be potential sources of infection are involved [134]. Emerging technologies can be considered and applied to ensure food safety, such as nanotechnology, cold plasma, ozone applications [135, 136]. For the food sector, the ultimate aim is to minimize the potential risk and supply safe food for consumers [134]. However, it is hard to achieve the goal under the context of the COVID-19 pandemic, and the food supply chain might be interrupted by the epidemics. Due to the burden brought by the cold chain-related virus contamination, China has suspended imports from many cold chain food manufacturers across various countries [131, 132], which will undoubtedly affect the smooth operation of the supply chain. For other countries, a similar situation might also exist. At present, a complete system covering early warning, tracing, and detection should be put forward in order to mitigate the COVID-19 outbreak. In the system, detection for SARS-CoV-2 should occupy a significant position for both populations and possible contaminated sources [137]. The Chinese government has required the overseas food exporters to strictly follow the regulatory guidelines issued by the Food and Agriculture Organization of the United Nations and the WHO [132]. It could be regarded as an effort to minimize the transmission risk, which will also help restore the normal supply chain. Other countries should take more actions in this aspect to help the reopen in the post-epidemic era.

## Implications for the global pandemic

Although the lockdown has stopped population movement across borders during the pandemic, continued global trade flows might contribute to the indirect transmission through shared contact of contaminated objects [75]. Except for China, Vietnam, and a few other countries, the possible contamination by the SARS-CoV-2 has not been detected regularly, which might cause new transmission. Moreover, because of differences in national conditions and culture, restriction measures implemented and the adherence of the public varied in different countries [138, 139], which is undoubtedly associated with the scale and duration of the epidemics in different regions. In the process of ending the pandemic, any possible risk source needs to be attached to importance. For many countries, considering the appearance of mutant viruses, the national governments must carry out credible plans to avoid the repeating spikes of SARS-COV-2 transmissions instantly. Besides, to reduce the impact, with the applications of effective and safe vaccines, many countries have released the “recovery roadmap” to ease the COVID-19 restrictions. In contrast, WHO has warned that the risks of returning to lockdown remain very real if countries do not manage the transition extremely carefully, and in a phased approach [140]. The coping strategies in China have significant implications for the world, especially for those countries urging to end the pandemic and ease restrictions. All countries need to evaluate risk carefully and make the right decision to respond to COVID-19 challenges.

## Conclusions and perspectives

We have discussed the possible roles of the cold chain through the comprehensive analysis for the cold chain-related epidemics in China, and similar examples in other countries, the roles of the cold chain need to be paid attention. The SARS-CoV-2 virus may contaminate foods during the production process, and a complex farm-to-table process augments the contamination risk. The cold chain supplies the virus with a favorable condition to survive during transportation. At the receiving end, those workers who contact the contaminated cold chain directly and repeatedly face the risk of infection. The transmission will undergo the process of “thing to human” and “human to human,” and indirect transmission might be the possible pathway causing the epidemics. A series of targeted prevention and control measures must be considered to face the challenges. The Chinese experience has provided a valuable reference for the world to respond to the global pandemic.

In brief, to end the pandemic earlier, the risk factors for SARS-CoV-2 transmission need to be recognized and handled continually and correctly, including the cold chain-related. Meanwhile, more research needs to be conducted to explore further the specific role of the cold chain in the outbreak of COVID-19.
